# Trisomy 21-associated increases in chromosomal instability are unmasked by comparing isogenic trisomic/disomic leukocytes from people with mosaic Down syndrome

**DOI:** 10.1371/journal.pone.0254806

**Published:** 2021-07-20

**Authors:** Kelly Rafferty, Kellie J. Archer, Kristi Turner, Ruth Brown, Colleen Jackson-Cook

**Affiliations:** 1 Department of Human & Molecular Genetics, Virginia Commonwealth University, Richmond, Virginia, United States of America; 2 Department of Pathology, Virginia Commonwealth University, Richmond, Virginia, United States of America; 3 Division of Biostatistics, College of Public Health, The Ohio State University, Columbus, Ohio, United States of America; 4 Department of Psychiatry, Virginia Commonwealth University, Richmond, Virginia, United States of America; IGBMC/ICS, FRANCE

## Abstract

Down syndrome, which results from a trisomic imbalance for chromosome 21, has been associated with 80+ phenotypic traits. However, the cellular changes that arise in somatic cells due to this aneuploid condition are not fully understood. The primary aim of this study was to determine if germline trisomy 21 is associated with an increase in spontaneous somatic cell chromosomal instability frequencies (SCINF). To achieve this aim, we quantified SCINF in people with mosaic Down syndrome using a cytokinesis-blocked micronucleus assay. By comparing values in their isogenic trisomic/disomic cells, we obtained a measure of differences in SCINF that are directly attributable to a trisomy 21 imbalance, since differential effects attributable to “background” genetic factors and environmental exposures could be eliminated. A cross-sectional assessment of 69 people with mosaic Down syndrome (ages 1 to 44; mean age of 12.84 years) showed a significantly higher frequency of micronuclei in their trisomic (0.37 ± 0.35 [mean ± standard deviation]) compared to disomic cells (0.18 ± 0.11)(P <0.0001). The daughter binucleates also showed significantly higher levels of abnormal patterns in the trisomic (1.68 ± 1.21) compared to disomic (0.35 ± 0.45) cells (P <0.0001). Moreover, a significant Age x Cell Type interaction was noted (P = 0.0113), indicating the relationship between age and SCINF differed between the trisomic and disomic cells. Similarly, a longitudinal assessment (mean time interval of 3.9 years; range of 2 to 6 years) of 18 participants showed a mean 1.63-fold increase in SCINF within individuals over time for their trisomic cells (P = 0.0186), compared to a 1.13-fold change in their disomic cells (P = 0.0464). In summary, these results showed a trisomy 21-associated, age-related increase in SCINF. They also underscore the strength of the isogenic mosaic Down syndrome model system for “unmasking” cellular changes arising from a trisomy 21 imbalance.

## Introduction

Down syndrome (OMIM 190685) is the most common cytogenetic condition seen in humans [1]. Although the chromosomal etiology of Down syndrome (a trisomic imbalance for chromosome 21) has been known for decades [2], the biological basis for how this trisomic imbalance results in the constellation of over 80 phenotypic findings that have been reported in people with this condition remains largely unclear [3–6]. In addition to the congenital traits that typically lead to the diagnosis of this condition in infancy or early childhood, individuals with Down syndrome also acquire additional health conditions, which include (but are not limited to) thyroid disease, alopecia, immune system abnormalities, osteoporosis, premature menopause, obstructive sleep apnea, hearing impairment, cataracts, osteopenia/osteoporosis, and Alzheimer-like dementia [7–11]. As people with Down syndrome are now living to older ages (mean life expectancy age of 57.8 years for females; 61.1 years for males [12]), the profile of acquired co-morbidities related to a trisomy 21 imbalance continues to evolve, along with the recognition of a need for biomarkers to facilitate diagnosing people who are most at risk for developing these co-morbidities.

One approach for improving our knowledge of factors contributing to the biological changes that arise with age in people with Down syndrome is to compare values between trisomic and disomic cells. Acquired chromosomal instability represents one subset of biomarkers associated with/related to acquired health conditions and aging in the general population [13], but there is a paucity of studies in which spontaneous acquired somatic chromosomal instability frequencies (SCINF) have been evaluated in people with Down syndrome. In the few previous reports, most investigators have observed increased SCINF in the buccal mucosa cells [14, 15] or lymphocytes [16–19] of people with Down syndrome compared to the levels seen in healthy, age-matched controls. However, genetic (e.g., genes that might contribute to an increased propensity to have acquired somatic cell instability) and environmental background differences between the different individuals in the trisomic and control groups have limited the investigators’ ability to directly attribute the observed changes to influences reflective of a trisomic imbalance [20].

**Fig 1:**
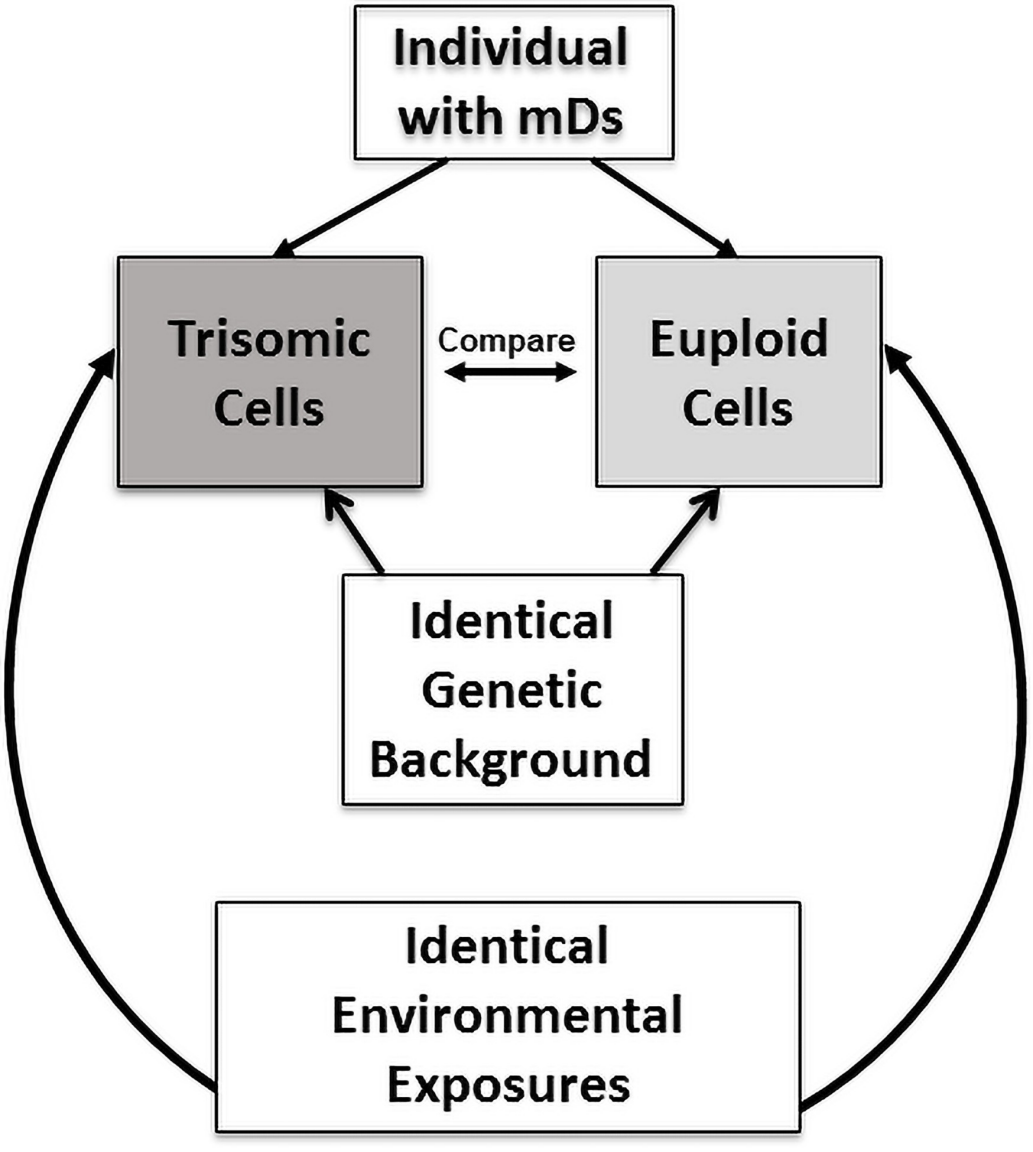
Isogenic trisomic-disomic mosaic Down syndrome study design. An individual with mosaicism for trisomy 21 has both trisomic and disomic cells that originated from a single zygote as a constitutional finding. Thus, these trisomic and disomic cells have identical genetic backgrounds (except for the trisomy 21 imbalance) and identical environmental exposures. By measuring trisomic compared to disomic cellular attributes, individual genetic “background” variation is eliminated to allow for direct assessments of trisomy 21-specific influences.

One approach to tease apart the cellular effects directly attributable to a trisomic imbalance is to study people with mosaicism, since these individuals have both trisomic and disomic (normal) cells that differ only for the presence (or absence) of an additional chromosome [21, 22]. Importantly, this “mosaic” study design approach not only removes the confounding effects of inter-individual differences due to total genetic make-up, but also controls for the effects of environmental influences, since the trisomic and disomic cells in people with mosaicism share identical exposure histories (Fig 1). The primary aims of this study were to: (1) determine if there are differences in SCINF between the isogenic trisomic and disomic cells obtained from people with mosaicism for a trisomic imbalance for chromosome 21; and (2) determine if the SCINF in the trisomic and/or disomic cells of people with mosaicism are influenced by age.

**Fig 2:**
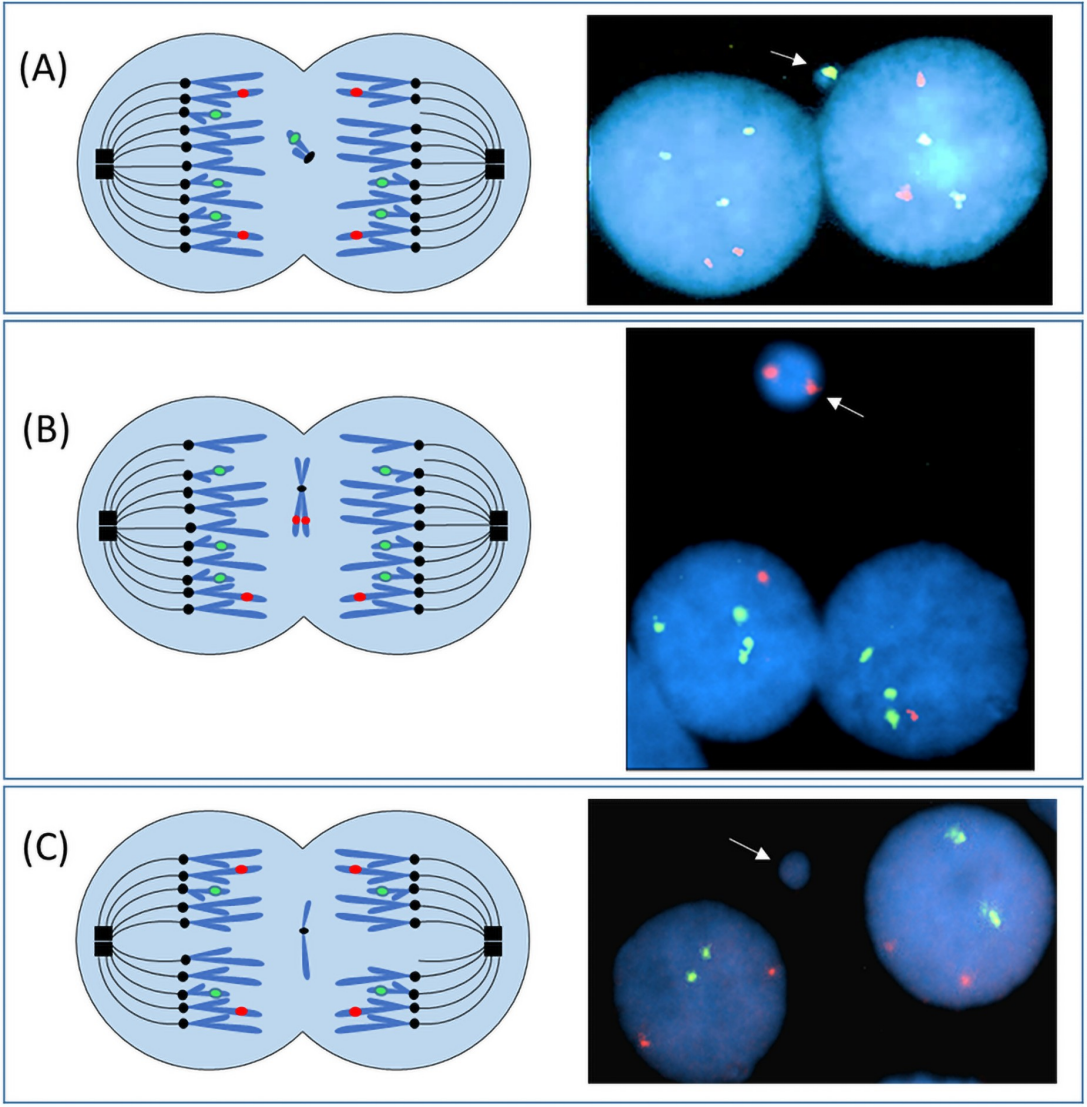
Cytokinesis-blocked micronucleus assay to quantify somatic cell instability frequencies in the trisomic compared to disomic cells from people with mosaicism for a trisomy 21 imbalance. This diagram illustrates one mechanism (chromosome or chromatid lagging) whereby micronuclei can form. (A) During the metaphase of a mitotic division one chromatid from chromosome 21 fails to attach to the spindle fibers. As a result, this chromatid lags behind during the anaphase migration and fails to segregate to the spindle poles (left diagram). Following karyokinesis, the laggard chromosome could be excluded from the daughter cell nuclei and become enclosed in a micronucleus. In the right photomicrograph of a trisomic cell, at least a portion of chromosome 21 was excluded into a micronucleus (white arrow). Only 2 signals for the chromosome 21 probe are present in the right daughter nucleus (loss of a chromosome 21 signal) compared to 3 signals that are present in the left daughter nucleus (RUNX1 probe [21q22; green]; RUNX1T1 probe (8q22; red)]. In panel (B) trisomic binucleates (3 signals for the chromosome 21 probe) are shown (illustration on left; photomicrograph on right) that had loss of one replicated chromosome 8 (both sister chromatids) into a micronucleus, resulting in daughter cells that each had a monosomic imbalance for chromosome 8. (C) A disomic binucleated cell (both primary nuclei have 2 signals for the chromosome 21 and chromosome 8 probes) has a single micronucleus that does not contain chromatin for either the RUNX1 (21q22) or the RUNX1T1 (8q22) loci.

A robust method to score SCINF is the cytokinesis-blocked micronucleus assay, which provides information about the presence of chromosomal findings with minimal influence(s) attributable to *in vitro* selective growth pressures [23]. This assay also allows for the assessment of a large number of cells, thereby enabling one to detect findings that may be present in low frequencies. Micronuclei are cytological structures that contain chromatin (from one or more chromosomes) that was not incorporated into the primary nucleus (Fig 2). A micronucleus can arise from a variety of mechanisms, including (but not limited to) those that occur: (1) prior to mitosis (acentric fragments; extrachromosomal fragments; replicative stress); (2) during mitosis (spindle checkpoint perturbations; microtubule alterations; defects in cohesion; centrosome dysfunction; pericentromeric alterations; kinetochore/centromere sub-functionality); (3) post-karyokinesis (chromosome bridges; nuclear envelope assembly defect(s); cell fusion after a multi-polar mitosis); or (4) in interphase (chromatin extrusion/budding [may involving double minutes]; disruption/rupture of the chromocenter or nuclear envelope; or nucleolar aggresome protrusions) [24]. Micronuclei are a biomarker for DNA damage, aneuploidy, and hypermutation [13, 23]. Increases in micronuclei frequencies have been associated with environmental exposures, aging, and several health conditions, including (but not limited to) cancer, diabetes mellitus type 2, and neurodegenerative diseases [13]. To our knowledge, this is the first study of SCINF in individuals with mosaicism for trisomy 21, and also the first to directly compare SCINF in isogenic trisomic and disomic cells via a cross-sectional and longitudinal study design.

## Materials and methods

### Ethics statement

This research involving human subjects was approved by the Virginia Commonwealth University Human Subjects’ Institutional Review Board (IRB # HM179 CR3). Written documentation of informed consent/assent was obtained from all study participants, with parental informed consent being obtained for assenting children, as well as adults who demonstrated intellectual disability levels that might compromise their ability to provide fully informed consent.

### Study participant ascertainment

A total of 69 participants with mosaic Down syndrome were ascertained through their membership/participation with the International Mosaic Down Syndrome Association. The only selection criterion was that the study participant have a chromosomally confirmed diagnosis of mosaicism for a trisomic imbalance for chromosome 21. After giving their informed consent/assent, peripheral blood specimens were collected for each study participant using venipuncture, which was performed by a trained health care provider/phlebotomist. A subset of these probands (n = 18) provided at least 2 blood specimens for this study (collected over at least a 2-year time span). This subset allowed for an additional, longitudinal assessment of SCINF of the isogenic trisomic/disomic cells within individuals.

### Quantitation of chromosomal instability

SCINF were quantified using the cytokinesis-blocked micronucleus assay [25, 26]. To evaluate micronuclei frequencies, leukocytes from peripheral blood specimens were collected using Histopaque-1077 (Sigma) and established in culture according to our adaptation of the protocol of Thomas, et al. [14]. Following mitogenic stimulation using phytohemaglutinin (PHA), lymphocytes were arrested at cytokinesis by adding Cytochalasin B (3.0 μg/ml; Sigma) to the cell suspension 44 hours after culture initiation. At 72 hours, cells were harvested as previously reported [27]. Briefly, this harvest included incubation in a hypotonic solution (0.075 M KCl) for 10 minutes, followed by fixation (three times using a 3:1 methanol: acetic acid solution). Slides were prepared following standard methods as previously described [27].

#### Micronuclei visualization and fluorescence in situ hybridization (FISH)

To distinguish between trisomic and disomic nuclei, a “test” probe targeting the RUNX1 locus (21q22) (Abbott Molecular) was hybridized to interphase cells, along with a differentially colored “control” probe for the RUNX1T1 locus (8q22) (Abbott Molecular). This dual color probe set has been validated in our CLIA and CAP approved cytogenetics laboratory and consistently shows specificity and sensitivity values of 0.99 or higher. Probe hybridization and visualization was achieved using the manufacturer’s fluorescence in situ hybridization (FISH) protocol (Abbott Molecular). Briefly, the DNAs in both the specimens and probes were co-denatured at 73°C for 2 minutes. Following denaturation, the slides were placed in a humidified chamber and hybridized at 37°C overnight. Non-specific binding of probes was removed by washing in a 0.4X SSC/0.3% NP-40 solution at 73°C for 2 min, followed by a 2 min wash at room temperature (in a 2X SSC/0.1% NP-40 solution). To visualize the binucleated cells and micronuclei, the chromatin in the nuclei and micronuclei was counterstained with DAPI II/antifade (Abbott Molecular).

#### SCINF scoring

An Axioskop (Zeiss) equipped with single- and triple-band pass filters was used to score the slides. The percentage of trisomic and disomic cells present in each study participant’s blood specimen was determined by randomly scoring 1000 interphase cells. The slides were then scored a second time in order to identify micronuclei associated with binucleated cells. The protocol used for recognition of a micronucleus/micronuclei followed the criteria established by Fenech [28]. A total of 1000 binucleates were scored for the micronucleus frequency assessments, with each binucleate being categorized as trisomic or disomic according to the chromosome 21 probe signal count. If at least one of the binucleated cells had 3 signals for the chromosome 21 probe, or if the combined number of probe signals was equivalent to trisomic expectations (i. e. one daughter cell with 2 signals and one with 4 signals [for a total of 6 signals (3+3) across both post-replication phase cells]), this cell was categorized as a trisomic cell. When scoring each cell, all focal planes were viewed to ensure the detection of all signals in the three-dimensional nuclei. Binucleated cells that did not have clear borders or that were overlapping were excluded from the analysis. Each micronucleus associated with the binucleates was categorized as follows based on the probe signals present: (1) positive for chromatin from 21q22 (21+ micronuclei); (2) positive for chromatin from 8q22 (8+ micronuclei); (3) positive for chromatin from both 21q22 and 8q22 (21+, 8+ micronuclei); or (4) negative for chromatin from the targeted loci on 21q22 and 8q22 (21-, 8- micronuclei). The relative proportion of micronucleated trisomic cells was calculated by dividing the number of trisomic cells having at least one micronucleus by the percentage of trisomic cells present in the blood specimen; likewise, the relative proportion of micronucleated disomic cells was calculated by dividing the number of disomic cells having at least one micronucleus by the percentage of disomic cells present in the specimen.

**Fig 3:**
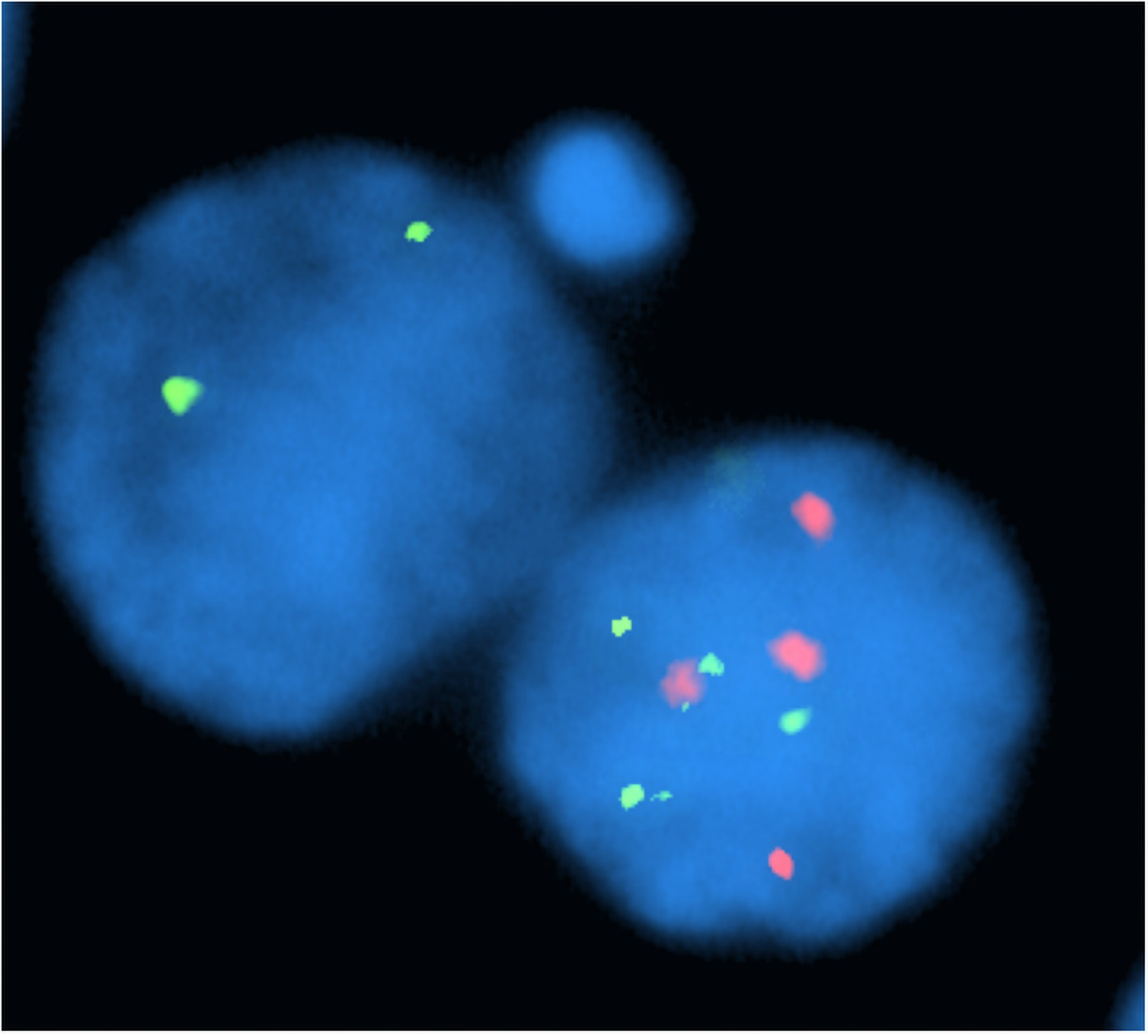
Assessment of imbalances for the RUNX1 (21q22) and RUNX1T1 (8q22) loci in daughter binucleates from micronucleated cells. This trisomic cell (total of 6 signals for chromosome 21; 4 signals for chromosome 8 [post-replication]) was categorized as “atypical” since the “daughter” binucleate pattern showed an imbalance that: (a) does not appear to result from the micronucleus formation (the micronucleus is 8q22-; 21q22-); and (b) arose from malsegregation events involving both chromosome 21 (pattern of 4 signals [right] and 2 signals [left]) and chromosome 8 (4 signals [right] and zero signals [left]).

Comparisons of chromosomal instability levels in the isogenic trisomic/disomic cells were also completed for the “primary” [also called daughter] binucleates (versus the micronuclei). This nuclear analysis was completed for 42 of the 69 probands (all specimens collected from 2017 to 2019]). Cells were categorized as “atypical” if they had an abnormal daughter binucleate probe signal pattern that: (1) did not appear to be directly related to the micronucleus formation; and (2) contained an imbalance that could not result from a single mitotic malsegregation event (i.e. imbalances involving both chromosome 21 and chromosome 8) (Fig 3).

### Statistical analyses

#### Cross-sectional analyses

A paired t-test was used to assess the relative proportion of micronuclei in trisomic compared to disomic cells. For trisomic and disomic cells, we tested whether the proportion of micronuclei containing chromatin from chromosome 21 was more frequent than what would be expected by chance. We used a z-test for proportions with the null proportion for trisomic cells of 0.064 (3/47) and the null proportion for disomic cells of 0.043 (2/46). Typically, the micronucleus frequency is a discrete count outcome that is highly skewed [29]. Therefore, to determine whether the frequency of micronuclei in trisomic cells is influenced by age, a Poisson regression model was used. A Poisson regression model was also fit to determine whether the frequency of micronuclei in trisomic cells, and separately the frequency of micronuclei is disomic cells, differed by sex. To determine if a potential age effect differed between trisomic and disomic cells, a mixed effects Poisson regression model was fit that included age, cell type (trisomic vs disomic), and their interaction as fixed effects, and patient as a random effect. Similarly, to determine whether the association between sex and micronuclei frequency differed between trisomic and disomic cells, a mixed effects Poisson regression model was used that included sex, cell type (trisomic vs disomic), and their interaction as fixed effects, and patient as a random effect.

To determine if there was a difference in the frequency of the binucleates showing an atypical (abnormal) probe signal pattern in the trisomic compared to disomic daughter nuclei, a mixed effects Poisson regression model was fit, using cell type (trisomic vs disomic) as a fixed effect and patient as a random effect.

#### Longitudinal analyses

To promote normality for analyses of the skewed longitudinal micronuclei data, a square root transformation was performed. A mixed effects linear model was evaluated on the transformed data to determine if the relative proportion of micronuclei increased over time, with this model having age as the fixed effect and subject as a random effect. Models were fit independently for trisomic and disomic cells. Similarly, to determine if changes in SCINF over time differed by sex, a mixed effects linear model was fit, with age, sex, and their interaction being identified as fixed effects, and subject as a random effect (models were fit independently for trisomic and disomic cells). Lastly, a linear regression model was used to determine if there was a significant association between the difference in the percentage of trisomic cells in the longitudinal blood specimens (Timepoint 1 minus Timepoint 2) and the micronucleus frequency at Timepoint 1.

## Results

### Study participant characteristics

The 69 study participants, all of whom had mosaicism for a trisomic imbalance for chromosome 21, included 29 males (42.0%) and 40 females (58.0%). The participants’ ages ranged from 1 to 44 years, with an overall mean of 12.84 years of age (Table 1). The average percentage of trisomic cells in the 69 participants with mosaicism was 33.95%, with values ranging from 4.8% to 96.0% trisomic cells (Table 1).

**Table 1:** Comparison of micronucleus frequencies in isogenic trisomic and disomic cells from 69 study participants with mosaicism.

Finding	Average (Standard Deviation)	Range	P Values
Age at specimen collection	12.84 (10.52)	1 to 44 years	
Cells with MN	20.23 (8.59) per 1000 binucleates	7 to 40 per 1000 binucleates	
Percent of trisomic cells in the blood specimens	33.95% (32.91%)	4.8% to 96.0%	
Relative proportion of cells with MN^1^			
Trisomic	0.37 (0.35)	0 to 1.51	**<0.0001**
Disomic	0.18 (0.11)	0 to 0.71	
Relative proportion of cells with atypical abnormal nuclear probe signal patterns^2^			
Trisomic	1.68 (1.21)	0.02 to 4.69	**<0.0001**
Disomic	0.35 (0.45)	0.0 to 1.98	

^**1**^Number of MN (micronuclei) in trisomic (or disomic) cells/percentage of trisomic (or disomic) cells

^**2**^Number of abnormal, atypical nuclei in trisomic (or disomic) cells/percentage of trisomic (or disomic) cells

### Trisomic compared to disomic SCINF frequencies

**Fig 4:**
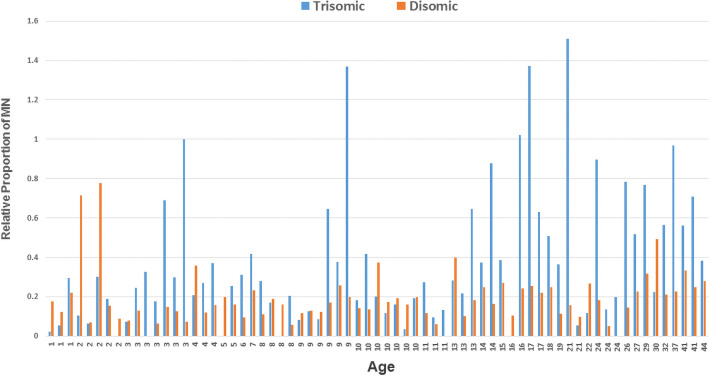
Relative proportions of trisomic and disomic cells containing micronuclei in the cross-sectional study. The data from the isogenic cells of the 69 participants who have mosaicism are ordered by age (youngest on left; oldest on right). The relative proportion of trisomic cells having micronuclei is shown with blue histograms, while the relative proportion of disomic cells is shown with orange histograms. The trisomic cells showed significantly higher levels of micronuclei than the disomic cells (P = <0.0001).

The results of a paired t-test showed that the relative proportion of micronucleated trisomic cells was significantly higher than the relative proportion of micronucleated disomic cells (P = <0.0001) (Table 1 and Fig 4). The frequency of micronuclei was significantly associated with age for the trisomic cells (P = 0.0011). Furthermore, when fitting the mixed effects Poisson regression model, a significant Age x Cell Type interaction was noted (P = 0.0113), indicating that the relationship between age and micronuclei frequency differed between the trisomic compared to disomic cells (Table 2). However, no association between sex and micronuclei frequency was noted in the trisomic (P = 0.1565) or disomic (P = 0.3605) cells. Moreover, the results of the mixed effects Poisson regression model showed no significant interaction for the Sex × Cell Type term (P = 0.6381) (Table 2). The increase in micronuclei frequencies in the trisomic cells was not limited to micronuclear events involving chromosome 21, since 81.0% of the micronuclei observed in the trisomic cells (compared to 85.5% in the disomic cells) did not contain chromatin from the 21q22 region interrogated in the probe set. However, micronuclei containing chromatin from chromosome 21 were observed more often than expected by chance in both the trisomic and disomic cells (18.99% of trisomic cells compared to chance expectations of 6.4% [3 of 47]; P<0.0001; 13.75% of disomic cells compared to chance expectations of 4.3% [2 of 46]; P = 0.0001).

**Table 2:** Mixed effects Poisson regression models to assess factors associated with micronuclei in people with mosaicism for a trisomic imbalance of chromosome 21.

Model (Predictive Variable)	Estimate	Std Error	z value	Pr(>|z|)
**Model Assessing Age, Cell type, and/or Age x Cell type interactions**				
(Intercept)	2.0534	0.0831	24.7004	<0.0001
Age at collection	0.0248	0.0046	5.4043	<0.0001
Cell Type	-0.1148	0.0891	-1.2886	0.1975
Age at collection By Cell Type	-0.0121	0.0048	-2.5320	0.0113*
**Model Assessing Sex, Cell type, and/or Sex x Cell type interactions**				
(Intercept)	2.3613	0.0754	31.3320	<0.0001
Sex (M)	0.0541	0.1150	0.4703	0.6381
Cell Type	-0.2198	0.0701	-3.1350	0.0017
Sex (M) by Cell Type	-0.1842	0.1095	-1.6827	0.0924

**Fig 5:**
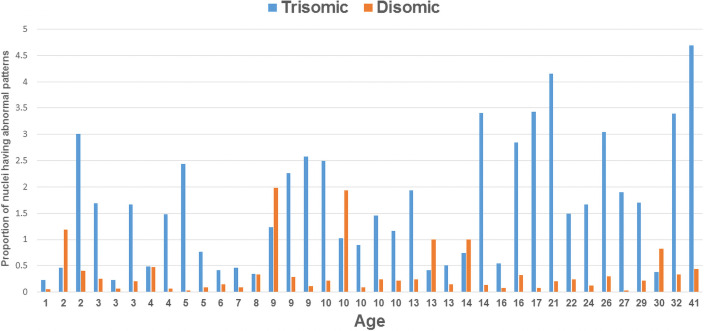
Relative proportions of trisomic and disomic interphase nuclei showing atypical abnormal RUNX1 and/or RUNX1T1 probe signal patterns. When compared to patterns in the disomic cells (orange histograms), the trisomic nuclei (blue histograms) showed significantly higher levels of atypical patterns (abnormal values involving more than a single chromosomal malsegregation event that were not clearly derived from the micronucleus formation) (p<0.0001).

In addition to evaluating the frequency of micronuclei between the trisomic and disomic cells, we also assessed the presence of chromosomal instability in the interphase nuclei, based on probe signal patterns for the targeted regions evaluated (8q22 and 21q21). A significant increase in the frequency of daughter nuclei showing abnormal probe signal patterns was observed for the trisomic compared to disomic cells (P <0.0001), suggesting that the chromosomal instability was not limited to the chromatin excluded into a micronucleus/micronuclei (Fig 5).

### Longitudinal assessment

To evaluate micronuclei frequencies in trisomic compared to disomic cells within a person over time, comparisons of micronuclei frequencies were completed for all 18 probands who provided more than one specimen. The average time between specimens was 3.89 years, with the range being 2 to 6 years. A significant increase in the relative proportion of trisomic cells having at least one micronucleus was observed at timepoint 2 compared to timepoint 1 (P = 0.0186), with a marginally significant value being noted for the disomic cells (P = 0.0464) (Table 3). However, the increase in SCINF over time did not differ by sex for the trisomic (P = 0.0693) or disomic (P = 0.3438) cells. A trend toward decreases in the percentage of trisomic cells over time was observed, (lower absolute values of trisomic cells noted at timepoint 2 for 14 of the 18 probands), but these values (percentage of trisomic cells at timepoint 1 versus timepoint 2) were not significantly different (P = 0.0731).

**Table 3:** Longitudinal assessment of micronuclei frequencies in participants with mosaic Down syndrome.

ID	Sex	Timepoint 1				Timepoint 2			
		Age	% Tri	RP MN Trisomic Cells^1^	RP MN Disomic Cells^2^	Age	% Tri	RP MN Trisomic Cells^1^	RP MN Disomic Cells^2^
21001	M	16	11.3	0.265	0.090	21	5.3	1.509	0.158
21004	M	8	41.9	0.310	0.138	13	41.2	0.218	0.102
21005	F	7	17.1	0.175	0.193	11	7.3	0.274	0.119
21007	M	24	17.7	0.621	0.146	29	18.2	0.769	0.318
21008	M	8	25.2	0.0	0.160	11	13.7	1.533	0.232
21009	F	9	18.0	0.333	0.207	13	12.4	0.645	0.183
21010	F	7	83.7	0.119	0.368	12	94.2	0.297	0.517
21011	F	7	21.4	0.140	0.140	10	12.3	0.163	0.194
21014	M	3	13.3	0.075	0.081	8	7.2	0.833	0.065
21017	M	4	18.4	0.272	0.123	8	15.1	0.199	0.130
21023	F	18	11.8	0.508	0.249	20	4.8	0.208	0.063
21028	F	35	21	0.238	0.241	41	7.1	0.563	0.334
21030	F	2	15.1	0.066	0.071	7	12.5	0.320	0.126
21039	F	3	13.4	0.299	0.127	6	8.9	0.787	0.176
21045	F	11	95.6	0.146	0.465	13	95.0	0.284	0.400
21062	M	7	18.3	0.164	0.061	10	10.3	0.194	0.201
21063	M	18	19.3	0.415	0.211	22	10.7	0.119	0.269
21080	M	22	9.1	1.868	0.254	24	7.8	0.897	0.184
**Avg**	**9M: 9F**	**11.6**	**26.2**	**0.334**	**0.185**	**15.5**	**21.3**	**0.545**	**0.209**

^1^RP MN Trisomic Cells = Relative proportion of trisomic cells that have at least one micronucleus

^2^RP MN Disomic Cells = Relative proportion of disomic cells that have at least one micronucleus

## Discussion

Aneuploidy has been posited to predispose somatic cells to acquired chromosomal instability [30–35], but this hypothesis has been challenging to test in humans [36]. Most of the extant literature assessing aneuploidy-related SCINF has been derived from cancer cell lines, the latter of which often have a cascade of biological/genetic changes that can confound the interpretation of the resultant data. Our study design, in which we directly compared spontaneous acquired SCINF values between trisomic and disomic cells that had identical genetic backgrounds (isogenic) and environmental histories (since they were both from the same person), allowed us to directly assess the impact of a trisomy 21 imbalance on the SCINF. We found that spontaneous SCINF were significantly higher in trisomic cells (compared to isogenic disomic cells) from people with mosaicism. We also found that SCINF were significantly associated with age and that the relationship between age and SCINF differed in the trisomic compared to disomic cells. Our observation of a differential influence of age between the trisomic and disomic cells is interesting; especially in light of the suggestion that people with Down syndrome show atypical aging (sometimes described as precocious aging) [8, 37].

A consistent association between age and sex has been observed for spontaneous micronucleus formation in healthy, chromosomally typical adults, with the sex influence being most pronounced for adults who are 40 years of age or older [38, 39]. Fewer investigators have studied spontaneous micronuclei frequencies in chromosomally typical children, with the results of these studies being varied, but generally showing weaker (or absent) associations with sex [40, 41]. Thus, the lack of an association between SCINF and sex in our cohort may reflect, at least in part, the age distribution of our cohort (mean age of 12.84 years [range of 1 to 44 years]).

We also noted that the majority of micronuclei from the trisomic (and disomic) cells (81.0%) did not contain chromatin from chromosome 21 (or at least not the 21q22 region evaluated in this study), suggesting that the SCINF may have a genome-wide impact. Nonetheless, chromosome 21 was present in micronuclei more often than expected by chance. Thus, the exclusion of chromatin into micronuclei may reflect, at least in part, some chromosome-specific attributes, rather than being purely stochastic events. Interestingly, Migliori et al. [42], who studied the contents of micronuclei from patients with Alzheimer disease, also found a preferential exclusion of chromosome 21 into micronuclei relative to levels observed in control individuals. Potential chromosome-21 specific attributes one could speculate might influence SCINF include (but are not limited to): alterations in centromere structure, methylation, and/or function; replication timing (i.e. late-replicating regions may have an increased propensity for sub-optimal spindle attachment leading to chromosome lagging), and size [43–45].

In addition to observing an increase in micronuclei frequencies, we also detected a significant increase in SCINF in the primary binucleates. This observation is consistent with the work of Santaguida, et al. [35], who showed that chromosome mis-segregation led to additional genomic instability. Similarly, Passerini, et al. [46], showed an association between a trisomic (or tetrasomic) complement and an increase in genomic instability (for trisomic cell lines involving different chromosomes that they developed using micro-cell mediated chromosome transfer methodology). Passerini, et al. [46] further suggested that the observed increase in trisomy-related genomic instability was attributable to replication defects and increased sensitivity to replication stress. Other investigators have also reported increases in SCINF in trisomic cells, with several of these investigators attributing the increase in chromosomal instability/mis-segregation to anaphase lagging of a chromosome or chromatid [32, 47–49].

**Fig 6:**
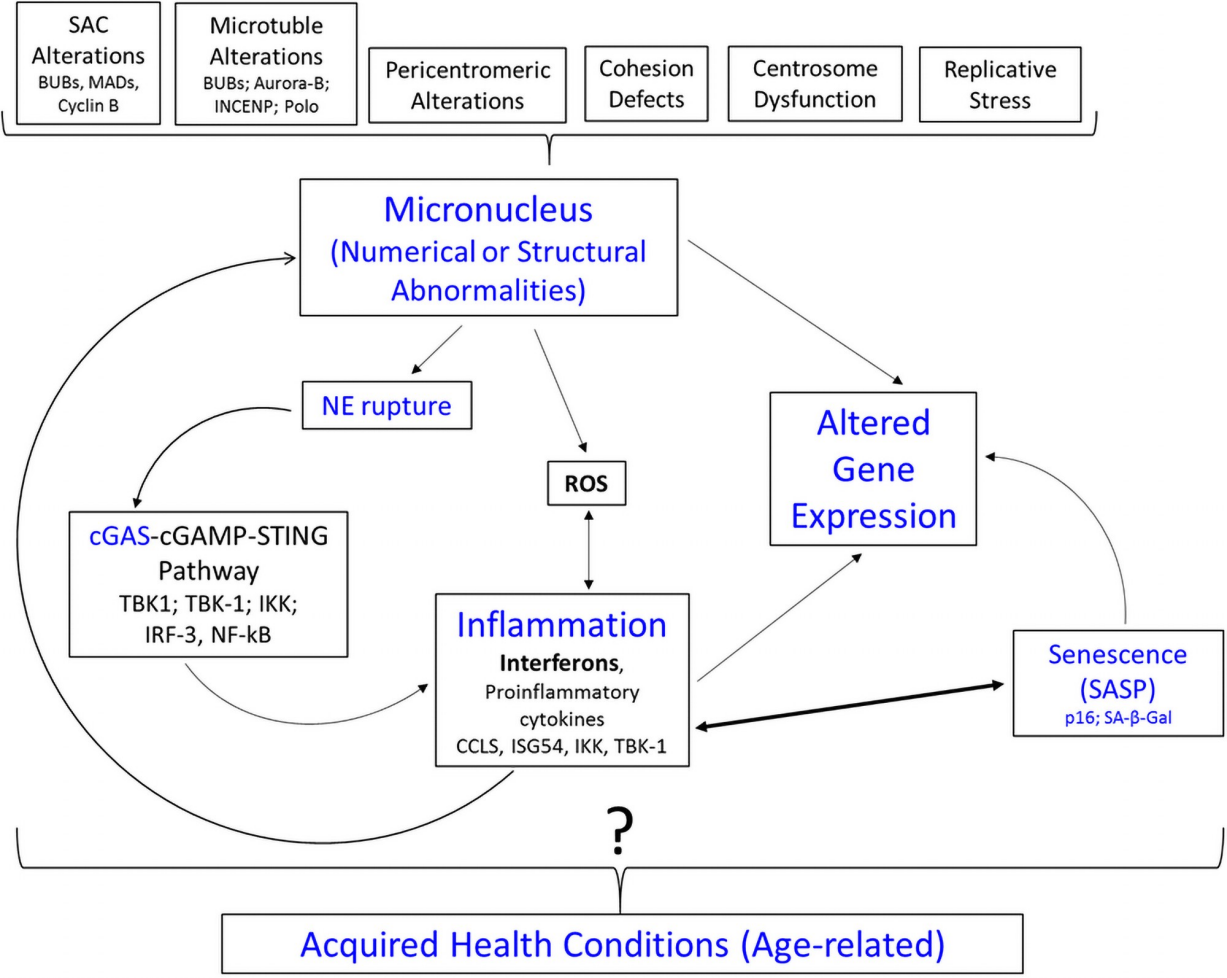
Hypothesized biological cascade related to increased micronuclei frequencies in cells with a trisomy 21 imbalance. Micronuclei can arise from a variety of mechanisms, including (but not limited to) factors that arise during mitosis (top of figure) (Guo et al., 2019). Due to nuclear envelope (NE) pores/gaps or rupture, micronuclei can be perceived as “cytoplasmic” DNA, triggering the cGAS-cGAMP-STING pathway, which in turn leads to the production of interferons. This “mark” targets the cells for senescence, the latter of which also contribute to inflammation (via SASP), thereby perpetuating the CIN-inflammation cycle. The micronuclei, either directly via genetic imbalance, or indirectly via inflammation and/or senescence, can contribute to alterations in gene expression. In turn, these alterations could contribute to the acquisition of age-related health conditions in people with Down syndrome or mosaic Down syndrome, but this conjectured relationship is not yet proven (as indicated by the?). SAC = Spindle Assembly Checkpoint; NE = Nuclear Envelope; ROS = reactive oxidative stress; SASP = Senescence Associated Secretory Phenotype.

Geneticists recently discovered that micronuclei (having nuclear envelope gaps) can be perceived as “cytoplasmic” DNA, leading to their detection by cyclic guanosine monophosphate (GMP)-adenosine monophosphate (AMP) synthase (cGAS). Upon binding, cGAS produces cyclic 2’3’GMP-AMP (cGAMP), which, in turn, binds and activates the stimulator of interferon genes (STING) protein, leading to type 1 interferons (IFNs) and proinflammatory cytokine production, as well as enhanced expression of ligands of natural killer cells and CD8+ T lymphocytes [50–56]. Type I IFNs can also induce DNA/chromosomal damage, thereby perpetuating a cycle of CIN-inflammation [55]. Activation of the cGAS-STING pathway, as well as whole chromosome instability, is also thought to promote cellular senescence and a senescence-associated secretory phenotype, leading to additional inflammation to perpetuate this cycle [34, 35, 53, 57]. The recognition that micronuclei stimulate innate immune surveillance via the cGAS-STING pathway has ushered a transition in thought regarding the role of micronuclei from being passive “reporters” of biologically relevant events to potential early “active” players in the initiation of cellular/genetic changes associated with aging and inflammation [13, 58]. The micronucleus/cGAS-STING pathway cascade is of particular interest in relation to traits acquired by people with Down syndrome, since several of these traits have been associated with perturbations in inflammatory pathways/immune dysfunction, as well as senescence [59–62] (Fig 6). Given that 4 of the 6 interferon receptor genes in humans are localized to chromosome 21 (including both type I IFNs), it is feasible, if not likely, that cells with trisomy 21 have an increased response to micronuclei [60, 63]. Similarly, a trisomic imbalance for the USP16 gene, which regulates ubiquitination of H2A-K119 and IKKs, could predispose people with Down syndrome or mosaic Down syndrome to have a “heightened” response to triggers leading to innate immune surveillance and/or senescence [59, 64, 65]. Moreover, the SOD1 gene (localized to 21q22.11), which metabolizes free radicals associated with reactive oxidative stress, provides an additional avenue for atypical cellular response(s) to SCINF in people with DS or mosaicism for trisomy 21 [66–68].

In agreement with the findings of our cross-sectional studies, our longitudinal studies also showed an increase in SCINF in the trisomic cells (1.63-fold increase). We also observed a marginal increase in SCINF over time for the disomic cells (1.13 fold increase). One could speculate that this latter observation reflects either: (a) “baseline” age effects; or (b) potential extrinsic (“bystander”) influences of cytokines/SASP that are primarily initiated by the trisomic cells (but also have potential to influence the disomic cells). Our longitudinal studies also showed a trend toward decreases in the percentage of trisomic cells at timepoint 2 (compared to timepoint 1) for the majority of our participants, which is an outcome one would predict if cells with chromosomal instability are being eliminated by immune surveillance, or cell-cycle arrested [36]. The observation of a decrease in the percentage of trisomic cells with aging in people with Down syndrome has also been noted by other investigators [69–72]. In addition to innate immune surveillance, factors that have been conjectured to contribute to acquired “loss” of trisomic cells in people with Down syndrome/mosaic Down syndrome over time have included differential growth rates and alterations in cell cycle kinetics [35, 36, 71, 73, 74]. While the majority of participants in the longitudinal study showed increases in SCINF, we also observed variation in these attributes from person to person, underscoring the fact that the consequences of aneuploidy are complex, multi-factorial, and can show variability [75].

### Study limitations and future directions

A limitation of this study is the age range of the participants, which did not include individuals beyond age 44. This age distribution reflects both: (a) the membership of the parent support group (IMDSA) through which the study participants were ascertained; and (b) the fact that cytogenetic testing methods were not optimized to detect mosaicism before the 1970s, making it quite challenging to identify people in their 50s, 60s or older who have a confirmed diagnosis of mosaicism. Another potential limitation regarding the generalizability of our findings is that one could view the mosaic cohort studied as a sub-population of people who have a predisposition to aneuploidy, since mosaicism has been noted to most frequently arise from 2 chromosomal malsegregation events (an initial meiotic event, followed by a second mitotic mis-segregation) [76, 77]. However, since the isogenic cells from the probands have identical background gene pools (other than the presence/absence of trisomy 21), any genetic predispositions to aneuploidy would be expected to also influence the segregation of chromosomes in the disomic cells. Thus, our observation of a clear difference in SCINF between the isogenic trisomic and disomic cells supports an association that is attributable to the trisomic imbalance.

One area for future studies related to our observation is to assess if there are associations between SCINF and health conditions acquired in people with Down syndrome/mosaic Down syndrome, and to determine if SCINF might represent an early step in a biological cascade that contributes to their development of inflammatory changes and co-morbidities (Fig 6). Another area for future study is to determine if there are differences in SCINF in the trisomic compared to disomic cells of people with mosaicism who were conceived from a meiosis I or meiosis II nondisjunction error, followed by a second mitotic error, compared to those seen in people with mosaicism due to a single mitotic event. Additionally, research using single cell sequencing and live cell tracking methodology, as well as studies targeting various biological components contributing to SCINF, could help to clarify the causes and outcomes of somatic chromosomal instability in people with a trisomic imbalance for chromosome 21.

In summary, the results of this study suggest that an increase in SCINF is an age-related, trisomy 21-associated cellular attribute. Importantly, the results of this study illustrate the value of the isogenic mosaic Down syndrome model system for teasing apart the impact of a trisomy 21 imbalance on the presentation of complex traits associated with Down syndrome.

## Supporting information

S1 Dataset (XLSX)
